# Multimodal physical exercise and functional rehabilitation program in oncological patients with asthenia. study protocol

**DOI:** 10.1186/s12912-021-00734-9

**Published:** 2021-10-22

**Authors:** Eduardo José Fernández-Rodríguez, Jesús González-Sánchez, Ana Silvia Puente-González, José Ignacio Recio-Rodríguez, Celia Sánchez-Gómez, Roberto Méndez-Sánchez, Juan Jesús Cruz-Hernández, María Isabel Rihuete-Galve

**Affiliations:** 1 Department of Nursing and Physiotherapy, University of Salamanca, Salamanca, Spain; 2 Medical Oncology Service, University Hospital Complex of Salamanca, Salamanca, Spain; 3 Institute of Biomedical Research of Salamanca (IBSAL), Salamanca, Spain; 4 Department of Developmental and Educational Psychology, University of Salamanca, Salamanca, Spain; 5 Department of Medicine, University of Salamanca, Salamanca, Spain

**Keywords:** Nursing, cancer, cancer related fatigue, Physical exercise, Functional rehabilitation

## Abstract

**Background:**

The increase in the survival of oncology patients include multiple side effects as cancer-related asthenia and dyspnea, which represents a serious health problem. An implementation of the conventional clinical practice, developed through multimodal physical exercise and functional rehabilitation program intervention, may be useful in controlling dyspnoea. This study aims to evaluate the effects of a multimodal exercise and functional rehabilitation program on fatigue, pain, functional capacity, and quality of life in cancer patients with cancer-related asthenia.

**Methods:**

This is a protocol for an experimental, prospective, randomised study using a parallel, fixed assignment scheme, with an experimental group and a control group in patients from the oncology hospitalisation unit at the Salamanca University Hospital Complex in Spain, using consecutive sampling to select 50 participants with oncological asthenia who are hospitalised at the time of inclusion. After the baseline evaluation, the participants will be randomised into two groups. Both groups will receive standard clinical practice care and the normal health education program at discharge, but in addition, the participants assigned to the experimental group will also complete a multimodal exercise and functional rehabilitation program lasting one month. The primary outcomes will be basic activities of daily living (Barthel Index) and degree of asthenia (FACT-An scale). Additionally, physical performance will be evaluated with the Short Physical Performance Battery (SPPB), as will the attention and executive functions (Trail-Making Test), fear/avoidance of movement (TAMPA scale), pain (VAS scale), and body composition (waist, hip, brachial, thigh, wrist, and ankle circumferences).

**Discussion:**

The results of this study may be translated to clinical practice, incorporating a specific autonomy recovery programme into comprehensive rehabilitation programmes of care for cancer patients with asthenia. The current study addresses to improve the conventional clinical practice by proposing a multimodal physical exercise and functional rehabilitation program intervention, which will be implemented by an interdisciplinary team, to try to improve the autonomy of cancer patients with cancer-related asthenia.

**Trial registration:**

ClinicalTrials.gov; ID: NCT04761289. (February 18, 2021). https://clinicaltrials.gov/ct2/show/ NCT04761289.

## Background

Nowadays, we observe an exponential increase in the five-year survival of patients with cancer, this is due to the improvement in preventive strategies as well as the greater knowledge about the oncologic pathology. Related to this higher life expectancy in cancer patients, an increase in the side effects of the treatments administered to surviving patients has also been observed [1]. These are mainly due to the increase in the lines of treatment employed. This increase produces a deterioration in the functionality and in the quality of life of the patients [2]. Some of these side effects include cancer-related asthenia, depression and dyspnoea [3]. Cancer-related asthenia is the most common symptom associated with cancer and its treatment, and it strongly impacts patient quality-of-life parameters [4]. However, this problem is still generally underestimated by many professionals in the field of oncology [5].

Patients with tumour asthenia often experience fatigue, which makes their daily lives difficult. In addition, the presence of fatigue is usually associated with alterations in body composition such as loss of muscle mass or an increase in visceral fat [6].

Among the intervention measures currently available to address this problem, the National Comprehensive Cancer Network (NCCN) expert panel considers educational measures, controlled therapeutic physical exercise, and energy conservation techniques as fundamental, always when undertaken within a complete functional rehabilitation program [7]. There is scientific evidence that asthenia can be better controlled after the implementation of non-pharmacological measures, especially controlled physical exercise [8, 9]. In addition, physical exercise has also been shown to mitigate the afore mentioned alterations in body composition that usually occur in cancer patients [10, 11]. Indeed, Fernández-Lao et al. showed that patients with breast cancer who performed a multimodal physical exercise program achieved a reduction in body fat and an increase in lean body mass [12].

The available scientific literature indicates that the efficacy of these physical exercise interventions to address cancer-related fatigue is increased when a psychological component is added, especially when a interdisciplinary (oncology, nursing, physiotherapy, occupational therapy, and medicine) biopsychosocial approach is taken [13, 14]. Thus, different aspects related to loss-of-function, pain, and fatigue associated with fear-avoidance disorders should also be explored and evaluated by testing the degree of kinesiophobia in patients with cancer [15]. The cognitive-behavioural model of fear of movement notes that patients with chronic pain or fatigue syndrome tend to avoid physical activity because they believe that this activity causes or exacerbates these symptoms; avoidance behaviour leads to even greater fear and symptoms, producing more pain or fatigue [15–17].

These factors can be extended to patients with cancer-related asthenia, which is why physical activity is important to help them avoid loss-of-function and reduced physical capacity [15]. Selection of the optimal intervention environment is based on clinical complexity and the patient’s ability to self-manage their situation. Several previous studies, including a meta-analysis of 14 randomised controlled clinical trials in breast cancer survivors with an intervention supervised by phone or email [18], have shown that supervised physical exercise interventions outside the healthcare provision environment, both in the community and at home, have had good results.

However, despite the potential benefits of such interventions, there is little evidence available on the possible effects that multimodal physical exercise and functional rehabilitation programs may have on patients with cancer-related asthenia outside healthcare settings. Thus, this present clinical trial was designed to address this research gap and to create empirical evidence regarding the effect of an interdisciplinary multimodal physical exercise and functional rehabilitation program intervention applied at the time of hospital discharge in patients with cancer-related asthenia.

From the above, we are proposing as an intervention a multimodal physical exercise and functional rehabilitation programme, implemented by an interdisciplinary team established by nurses, occupational therapists, physiotherapists, and specialist doctors, will improve the autonomy of patients with cancer-related asthenia. It would be a reeducation in the completion of the activities of daily living, so as to foment the recovery of personal autonomy. The findings will provide important insight into the development of an effective care model with a comprehensive rehabilitation programme that includes a specific autonomy recovery programme in oncological patients with asthenia. Taking into account the results obtained in our clinical practice in the Oncology Hospitalisation Unit at the University Hospital Complex of Salamanca (CAUSA), we consider important the implementation of specific clinical care protocols for this type of cancer patients, and its study through clinical trials.

## Methods/design

### Design and aims

This experimental, prospective, randomized, parallel-controlled clinical trial, with two arms of fixed assignment with an experimental and a control group, will be conducted during a year in the Oncology Hospitalisation Unit at the CAUSA. We will use consecutive sampling to select the participants with oncological asthenia who are hospitalised at the time of inclusion.

This is one of the clinical trials that are starting in our Unit, with an interdisciplinary care team, and similar design, patients and procedures with others, such us the recent study protocol published [19].

According to the background, the primary aim of this study is to assess the effects of a multimodal physical exercise and functional rehabilitation program on fatigue, pain, functional capacity, and quality of life in cancer patients with cancer-related asthenia. The objectives are focused to compare the effects produced by the implementation of a multimodal physical exercise and functional rehabilitation program, with an isolated intervention using standard clinical practice treatment.

### Sample/participants

Participants: The patients will be recruited from the Oncology Hospitalisation Unit at the CAUSA when they will be hospitalised. The Unit’s investigators will invite patients to participate in the study by explaining the details of the clinical trial, and they can then be included once they have given their verbal and written consent, and meet the following selection criteria: (A) Inclusion criteria: Participants must have a pathological diagnosis of an oncological disease, be over 18 years of age, be hospitalised at the time of recruitment in the Oncology Hospitalisation Unit at the CAUSA, having a score of 15–55 points on the Barthel Index (BI), having a score of 4 or more on the visual-analogue scale (VAS) for cancer-related asthenia and have signed an informed consent form indicating voluntary agreement to participate in the study. (B) Exclusion criteria: not having an adequate cognitive state to be able to comprehend and follow the orders provided (fewer than 23 points on the Mini Mental State Examination, MMSE), and having a haemoglobin level lower than 10 g/dL. (C) Withdrawal criteria: disease progression which brings the patient to a terminal situation or to death, and non-completion of the follow-up and final assessments.

**Fig. 1:**
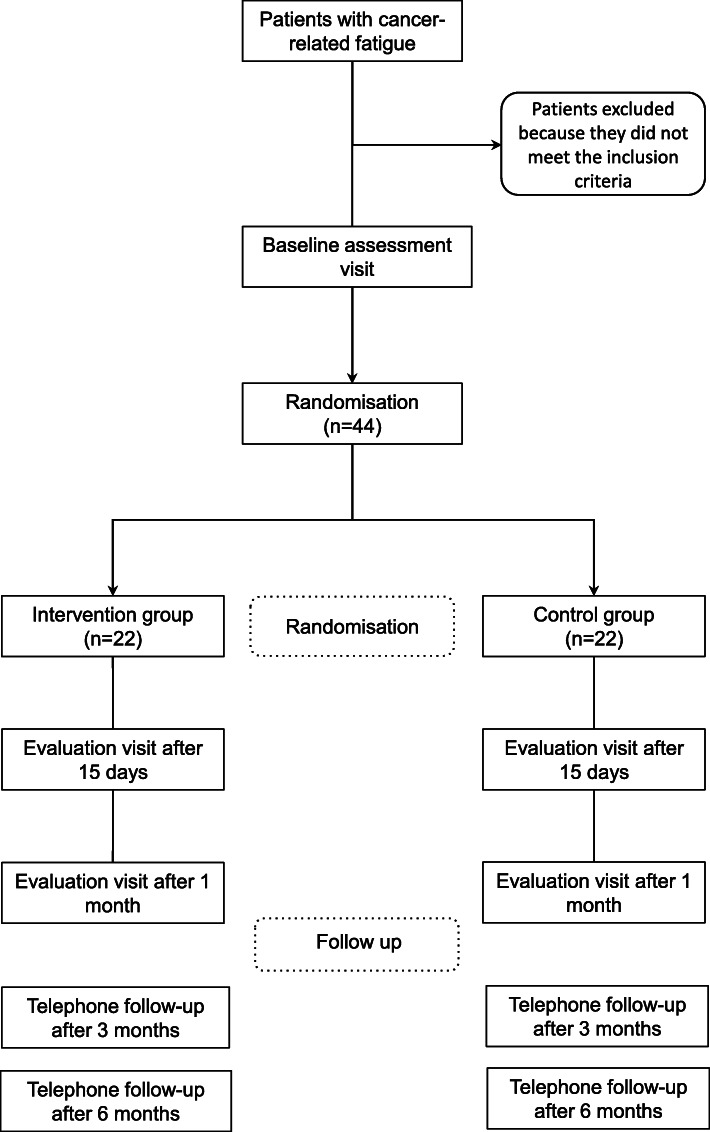
Study design. The enrolled participants will be randomly assigned to one of the study groups and will be assessed at three pre-specified time p

Assignment, randomisation and blinding: After recruitment, individuals will be assigned by a randomisation process to one of two study groups: Experimental Group or Control Group. Detailed in Fig. 1.

We will employ a simple randomisation process that consists of generating a table of random numbers of the same size as the estimated study sample with Microsoft Excel 2020 to assign subjects to each of the study groups. For even numbers, the subject will be assigned to the Experimental Group, and for odd numbers, the subject will be assigned to the Control Group. Randomisation, recruitment and assignment of subjects to each group will be performed by research staff not involved in the evaluations or interventions in each group, which will avoid potential bias in the study.

The participating subjects will not be blinded, due to the specific characteristics of the intervention in each group. However, the researchers responsible for the study measurements and statistical analysis will be blinded, with the intention of avoiding any contamination between groups, thus increasing the rigour of the study process.

Sample size: The sample size has been estimated based on the potential modification of one of the main outcome of the study—the score on the Barthel Index. For this calculation, we have considered the results obtained in a similar, where a modification of 17 points in the BI score was obtained, as a difference attributable to the intervention, considering this as the difference between the modification of the experimental group and the control group [20]. With these premises, accepting an alpha risk of 0.05 and a beta risk of 0.2 in a bilateral contrast, with 50 individuals (25 subjects in the experimental group and 25 in the control group, the statistical power was 90 % to recognize as statistically significant a change of 17 points between both groups in the BI score. The sample size was estimated using the programme EPIDAT 4.2.

### Procedures and data collection

#### Evaluations and study plan

Participants will be assessed at three points during the study: Firstly, the baseline assessment at the beginning, the second assessment after seven days, and a third final assessment, before the patients are discharged from hospital. The baseline assessment will be performed after recruitment and before randomisation and assignment of subjects to the corresponding group. This initial assessment includes the recording of the independent variables and the primary and secondary outcome variables of the study. Then, after randomisation, the corresponding intervention will begin to be applied to each group. Subsequently, the other further assessments will be carried out, one after seven days, considered as the follow-up assessment, and another at hospital discharge, considered as the final assessment. This can be seen in more detail in Table 1.

**Table 1:** Timeline and dimensions assessed at the time points

	STUDY PERIOD							
	**Enrolment**	**Allocation**	**Post-allocation**					**Close-out**
**TIMEPOINT**	***Month 1-2***	***Month 2-3***	***M4***	***M5***	***M6***	***M7***	***M8***	***M9-M12***
**ENROLMENT**:								
**Eligibility screening**	X							
**Database preparation**	X							
**Informed consent**		X						
**Allocation**		X						
**INTERVENTIONS**:								
***[Experimental Intervention]***								
***[Control Intervention]***								
**ASSESSMENTS**:								
***[BASELINE ASSESSMENT]***		X						
***[FOLLOW-UP ASSESSMENT]***								X
***[FINAL ASSESSMENT]***								X
***DISSEMINATION OF RESULTS***:								X

#### Description of the variables

The main variable will be the degree of dependence in performing the activities of daily living (ADL), as measured using the Barthel Index. The secondary variables will be cancer-related asthenia, attention and cognitive functions, health-related quality of life (HRQoL), pain, functional capacity, fear/avoidance of movement using the Tampa Scale for Kinesiophobia associated with Fatigue (TSK-F), and body composition. We will also record the following intervening variables from the patient clinical history: pathological diagnosis, number of treatment lines, and other sociodemographic and anthropometric data.

#### Tools employed in the evaluation of the variables

1. “Barthel Index, (BI)” [21]: to evaluate dependence. Measures physical disability with proven validity and reliability. It is easy to apply and to interpret. Useful for evaluating the patient’s functional independence in the activities of daily living (ADL). Scored from 0 to 100, it quantifies the individual’s degree of dependence.
2. “4th version of the Functional Evaluation of Cancer Therapy-Anaemia (FACT-An)” [22]: to evaluate cancer-related asthenia and the functional capacity. The FACT-An scale comprises the FACT-G fatigue subscale, plus seven non-fatigue items related to anaemia in cancer patients. In this study, we will use the version of FACT-G for cancer-related fatigue.
3. “Trail-Making Test (TMT)” [23]: to evaluate attention and executive functions. Useful for to assess attention, flexibility of thinking, and visuospatial ability. The global TMT comprises two parts: the TMT A measures attention, and the TMT B assesses processing speed and executive functions.
4. “EuroQol 5-D Questionnaire (EQ-5D)” [24]: to assess the health-related quality of life (HRQoL). It has been adapted and validated for use in the Spanish population.
5. “A Visual Analogue Scale (VAS)” [25, 26], to evaluate the pain. This will be assessed using the VAS as the most widely used pain assessment scale pain intensity is represented on a 10 cm line where 0 indicates the absence of pain and 10 represents the worst pain imaginable. The patient indicates the intensity of their pain; values less than 4 mean mild or mild–moderate pain; 4–6 indicate moderate–severe pain; and greater than 6 implies very intense pain.
6. “Short Physical Performance Battery (SPPB)” [27]: to evaluate Functional capacity. This test has been validated in the Spanish population for primary healthcare, and was specifically designed to predict disabilities, combines balance, gait speed, and the ability to get up from a chair and has been shown to predict adverse events, dependency, institutionalisation, and mortality.
7. The “Tampa Scale of Kinesiophobia–Fatigue (TSK-F)” [18, 28], was developed to evaluate the fear of movement related to fatigue/pain. It has been proven effective in oncology patients and those with chronic fatigue syndrome. We will use the model with 11 items (TSK-F-11).
8. Evaluation of body composition: by measuring the waist, hip, brachial, thigh, wrist, and ankle circumferences. Height and weight will be measured using a portable stadiometer (Seca, 222), calculating the average of two recorded measurements.

An individual data collection sheet will be used for each patient and recorded in a database specifically designed for this study.

#### Interventions

Conventional standard of care will be applied in both groups, explaining to each subject participating in the study the importance of compliance and adherence to the prescribed pharmacological treatment and the established guidelines personalised to their specific health needs. In the days prior to hospital discharge, they will also receive, as a part of the intervention, a health education programme with an informative talk and written guidelines, mainly focused at reinforcing and promoting an active and healthy lifestyle.

The difference of the intervention in the experimental group will be due to the fact that we will also implement the intervention with a multimodal programme of physical exercise and functional rehabilitation lasting one month. This will consist of:

1. A prescription for multimodal physical exercise. In the Oncology Hospitalisation Unit at the CAUSA, as in other studies already initiated [19], we will carry out the following design of a multimodal physical exercise programme. A supervised and structured program will be implemented in these patients for one month. This will consist of two short 15–20-minute sessions performed daily, one in the morning and one in the afternoon. The sessions will be structured according to the recommendations of the American College of Sports Medicine (ACSM) [29, 30], with an initial warm-up (2–3 min), the main section comprising the principal physical exercises (8–12 min), and a final cool-down and relaxation phase (5 min).

Parts of a standard session: The warm-up will consist of exercises designed to promote joint mobility and muscle activation. The main section will combine aerobic and balance exercises with low-load strength exercises targeting upper and lower quadrant muscle groups. The dosage and load will always be adapted according to patient evaluations performed beforehand and will follow a progression in difficulty with the aim of achieving an intensity at 50–75 % of each patient’s maximum heart rate or a perception of moderate effort on the Borg Scale. After 15 days of the program, the second evaluation will be carried out and the program may then be modified by adapting the load. Finally, the cool down will consist of gentle muscle stretching and relaxation exercises.

2. Activities of daily living re-education. Specific training designed to identify factors impeding the performance of the ADL will be conducted after the assessment and before the time of patient discharge from the hospital. This intervention will comprise three parts: (1) a direct ADL intervention carried out in situ in the hospital which will be generalisable to the patient’s everyday environment; (2) teaching of energy saving techniques (ESTs) based on the simplification of activities; and (3) advice on sleep hygiene measures for sleep disorders, as outlined in the specific NCCN guidelines on cancer-related asthenia [31].

3. Prescription of assistive products and environmental adaptations. Before the patient is discharged and after the baseline evaluation, we will evaluate if any support products that will favour their autonomy can be prescribed and at the time of hospital discharge and we will identify possible barriers in their home that could impair their autonomy.

#### Work plan and visit structure

Once the possible study candidates have been identified in the Oncology Hospitalisation Unit at the CAUSA, an interview with each individual will be organised to explain the purpose of the study to them and thereafter, invite them to sign the informed consent to their participation. Each participant will complete 3 planned evaluation visits. One at baseline, prior to randomisation, and two follow-up visits 15 days and 1 month after the first visit. The structure of all three 3 visits will be the same and each will last approximately one hour. In addition, there will be a telephone follow-up 3 and 6 months after the randomisation. The study variables will be evaluated during these visits, as specified in Table 1.

#### Baseline visit

This will be performed prior to patient discharge and ensuring compliance with the selection criteria. The evaluation will involve collection of all the study variables and completion of the questionnaires at baseline; the patient sociodemographic data, medical history, presence of comorbidities, and use of concomitant medications will also be recorded. At the end of this visit the patients will be randomly assigned to one of the two study groups. Participants included in the experimental group will then be explained the structure and organisation of the functional rehabilitation program during a specific visit planned for this purpose.

#### Follow-up visits at 15 days and 1 month

These visits will be identical to the baseline evaluation, except that the sociodemographic variables will only be collected at the baseline evaluation. The follow-up visits will be completed in the Clinical Teaching Assistance Unit in the Faculty of Psychology at the University of Salamanca, Spain.

### Data analysis

The statistical analysis will be carried out on an intention-to-treat basis. The characteristics of the population will be presented as means and standard deviations for continuous variables and as a frequency distribution for qualitative variables. To assess comparability of the two study groups at the baseline, the means between both groups will be compared using chi-square tests for qualitative variables and Student t-tests for qualitative variables. The effect of the intervention on the study variables will be evaluated using a repeated measures ANOVA with two study factors: time * group. Subgroup analyses will be carried out considering certain variables and/or categories from the baseline evaluation such as age or the initial FACT scale score. For hypothesis testing, an alpha risk of 0.05 will be set as the limit for tests of statistical significance. We will use SPSS software (version 23.0; IBM Corp., Armonk, NY) for all the statistical analyses.

### Rigour

This protocol study also follows the evidence-based recommendations of The SPIRIT 2013 Statement for the minimum content of a clinical trial protocol. And the design of the study also follows the evidence-based, minimum set of recommendations of the CONSORT 2010 Statement for conduct parallel-group randomized controlled trials (RCT), enabling readers to understand a trial’s design, conduct, analysis and interpretation, and to assess the validity of its results.

Data availability: Upon completion of the study, the data gathered herein will be made available to those investigators who request it through FAIRsharing of the repositories of research data at https://www.re3data.org/.

## Discussion

Cancer-related asthenia is considered the most common symptom associated with tumours and their treatment and is an essential factor in the functional deterioration of individuals with cancer [5]. There is growing interest in conducting clinical trials on the influence of physical exercise on the quality of life of cancer patients, although how these beneficial influences might affect the autonomy of patients in their daily lives remains to be addressed. However, these beneficial effects have been observed in daily clinical practice in patients with cancer-related asthenia in terms of physical improvements. Nonetheless, difficulties in generalising this physical improvement to occupational performance have also been noted [7–9].

As oncological clinicians, one of the biggest problems we face on a daily basis is that, although the acute patient symptoms will have been optimally resolved at the time of hospital discharge, there is still room for improvement in functional terms. With the planned follow-up described in this article, we intend to resolve this handicap by incorporating a prescription for specific rehabilitation methods for patients. We consider it essential to include ADL re-education techniques as part of the multimodal physical exercise rehabilitation methods [9].

Furthermore, in order to favour both an adequate intervention environment as well as self-management of situations by patients, our study proposes a supervised home-based intervention adapted to the specific situation of each patient with moderate cancer-related asthenia for implementation after their discharge from hospital. This meets the NCCN recommendations that better, more accessible physical exercise programs should be offered which patients are able to easily adhere to. In addition, they also suggest that the prescription of support products should be incorporated as these will contribute to achieving greater immediate functional improvement of individuals.

## Limitations

This study will follow all the recommendations of the CONSORT guidelines, but due to the nature of the intervention itself, the participants will not be blinded to the intervention. However, the researchers responsible for carrying out the study measurements in each evaluation and for the statistical analysis will be blinded.

### Dissemination plan

Dissemination of the study results will be carried out with the intention of ensuring maximum visibility. The results will be published in peer-reviewed open access scientific journals. There will be an initial publication of the primary results, with further publications of the secondary results planned. The results will also be presented at major national and international scientific conferences and seminars. Finally, the research team and the Oncology Hospitalisation Unit at the CAUSA will disseminate the results in social networks and other media.

### How potential changes in the study will be approached

Significant modifications to the protocol (such as change in the tools of evaluation, modifications to the selection criteria or to the interventions) will be communicated immediately to the bioethics committee.

## Conclusions

This study protocol aimed at assessing the influence of incorporating an interdisciplinary intervention, carried out by nurses, occupational therapists, physiotherapists, and specialist doctors in patients with cancer-related fatigue, with the aim of improving conventional clinical practice, and introducing a program of multimodal physical exercise and functional rehabilitation, which we consider essential in the follow-up of patients once they are discharged from hospital.

## Data Availability

Upon completion of the study, the datasets generated and/or analysed during the current study will be available to those investigators who request it through FAIRsharing of the repositories of research data at https://www.re3data.org/.

## Abbreviations

SPPB: Short Physical Performance Battery
NCCN: National Comprehensive Cancer Network
VAS: visual-analogue scale
MMSE: Mini-Mental State Examination
BI: Barthel Index
HRQoL: Health-related quality of life
TSK-F: Tampa Scale for Kinesiophobia associated with Fatigue
FACT-An: Functional Evaluation of Cancer Therapy-Anaemia
TMT: Trail-Making Test
EQ-5D: EuroQol 5-D Questionnaire
ACSM: American College of Sports Medicine
ADL: Activities of daily living
